# Post-Polymerization Modification of Poly(l-glutamic acid) with d-(+)-Glucosamine

**DOI:** 10.3390/molecules191219751

**Published:** 2014-11-27

**Authors:** Peter Perdih, Sašo Čebašek, Alenka Možir, Ema Žagar

**Affiliations:** National Institute of Chemistry, Laboratory for Polymer Chemistry and Technology, Hajdrihova 19, SI-1000 Ljubljana, Slovenia

**Keywords:** poly(L-glutamic acid), glucosamine, glycopolypeptide

## Abstract

Carboxyl functional groups of poly(L-glutamic acid) (PGlu) were modified with a D-(+)-glucosamine (GlcN) by amidation using 4-(4,6-dimethoxy-1,3,5-triazin-2-yl)-4-methylmorpholinium chloride (DMTMM) as a coupling reagent. The coupling reaction was performed in aqueous medium without protection of hydroxyl functional groups of D-(+)-glucosamine. Poly(L-glutamic acid) and GlcN functionalized polyglutamates (P(Glu-GlcN)) were thoroughly characterized by 1D and 2D NMR spectroscopy and SEC-MALS to gain detailed information on their structure, composition and molar mass characteristics. The results reveal successful functionalization with GlcN through the amide bond and also to a minor extent through ester bond formation in position 1 of GlcN. In addition, a ratio between the α- and β-form of glucosamine substituent coupled to polyglutamate repeating units as well as the content of residual dimethoxy triazinyl active ester moiety in the samples were evaluated.

## 1. Introduction

Carbohydrate moieties attached to a polymer backbone make the resulting copolymers applicable in various biomedical applications since carbohydrate moieties play a key role in many biological processes, e.g., cell–cell contacts, signal transmission, inflammation and recognition [1,2,3]. Thus, glycopolymers have been employed as macromolecular drugs [4], carbohydrate-based vaccines [5], carriers for drug [6,7] and gene [8] delivery, hydrogels [9], agents for MRI visualization of specific cell types [10] and as matrices for cell cultures [11,12,13]. Furthermore, synthetic glycopolymers were studied as antifreeze glycoprotein mimics [14]. 

Glycopolymers have been synthesized by polymerization of glycosylated monomers or by post-polymerization glycosylation of the precursor polymers [15,16]. Polymerization of glycosylated monomers was conducted by various techniques, *i.e.*, the controlled radical polymerization techniques [3,17,18,19,20,21] and ring-opening polymerization (ROP) of glycosylated α-amino acid *N*-carboxyanhydride (NCA) monomers [22,23,24,25,26,27,28,29,30,31].

The synthesis of glycopolymers by the post-polymerization modification approaches avoids the complex and tedious procedures required for glycosylated monomer synthesis and purification [3]. On the other hand, the post-polymerization strategies often suffer from incomplete glycosylation [23]. To circumvent these issues Deming and Kramer [32] reported highly efficient post-polymerization modification procedure for preparation of glycosylated poly(l-methionine). More often the click reactions are applied for glycosylation of the propargyl- or the azide-functionalized polymers with complementary modified carbohydrates. Various polymethacrylates were thus click-glycosylated [3,33,34]. Wu *et al.* [35] expanded the applicability of the azide-alkyne click chemistry to quantitative glycosylation of alkyne peripheral groups of dendrimers with azide-bearing sugars. The azide-alkyne, thiol-ene and thiol-yne reactions have been successfully applied also for efficient modification of polypeptides with carbohydrates [2,36,37,38,39,40,41,42,43,44,45,46].

If quantitative glycosylation is not the objective, the sugar functionality is often introduced into the polymer via amide bond formation [23]. The amine side-chain groups of polylysine were reacted with glycosyl lactones under basic conditions to attach the sugar moieties to polylysine via the amide bonds [47]. Various condensation agents were reported for modification of polymers with carbohydrate and other biologically interesting substituents. Activation of carboxyl functional group was achieved by applying carbodiimides [48,49], *N*-hydroxysuccinimide ester [50], pentafluorophenyl ester [34] or 4-(4,6-dimethoxy-1,3,5-triazin-2-yl)-4-methylmorpholinium chloride (DMTMM) coupling reagent. The DMTMM was applied to attach aminosugars to poly(acrylic acid), poly(methacrylic acid) [51], poly(L-glutamic acid) [52] and protein [49] scaffolds in good yields and with controllable degrees of substitution.

Amphiphilic glycopolymers were shown to self-assemble in water [38,53,54,55] and were for this reason studied for the application in RNA [56] and drug [57] delivery, and for construction of synthetic viral capsids [58]. Nanoparticles were formed from glycopolymers also by using phenylboronic acid moiety as reversible crosslinking agent [59]. These glycopolymer based nanoparticles were loaded with insulin and studied *in vitro* [60] and *in vivo* [61] for insulin delivery.

Well defined glycosylated acrylate block copolymers synthesized in a sequence-controlled fashion without post-polymerization modifications were prepared and successfully tested as competitive substrates for a C-type lectin [21]. In another study, a series of glycosylated methacrylamides were prepared [34]. Glycopolymers were synthesized with varying degrees of carbohydrate moiety substitution and with variable linker lengths between the carbohydrates and the methacrylamide backbone [34]. By applying a post-polymerization modification strategy, a galactose derivative was incorporated into the poly(methacrylamide) scaffolds. These glycopolymers were tested in bacterial-toxin and peanut agglutin inhibition experiments to reveal the impact of the glycopolymer characteristics on their inhibition potency [34]. 

Since the biocompatibility and biodegradability of glycopolymers are important criteria for their applicability in biomedical applications, synthetic glycopolypeptides are expected to be more appropriate for biomedical applications than glycosylated polymers bearing robust non-degradable carbon–carbon bonds in the backbone [2]. Recently, Mildner and Menzel [52] reported on the synthetic procedure for facile post-polymerization modification of poly(l-glutamic acid) (PGlu). PGlu was modified in water by coupling the amino sugar to the PGlu carboxylic functional groups in the presence of DMTMM as a coupling reagent. The resulting set of amino sugar modified polypeptides was characterized by ^1^H-NMR and FTIR and the secondary structures of the obtained glycopolypeptides in solution were investigated by circular dichroism spectroscopy in the media with different pH values. 

DMTMM has been applied as a coupling reagent for the preparation of glycopolymers [49,51] as well as for modification of carboxyl group-containing polysaccharides [62] and poly(l-glutamic acid) (PGlu) [63] with various amines in aqueous solution. Earlier, DMTMM was applied to modify poly(acrylic acid) with a wide range of amines bearing alkyl, hydroxyl, sulfonic acid or perfluoroalkyl groups [64]. DMTMM was thus shown to selectively promote amide bond formation in aqueous solution in the presence of non-protected hydroxyl and some other functional groups [51,64].

Research in our group is oriented toward the synthesis of polymeric carriers for delivery of biopharmaceuticals in the form of nanoparticles prepared by polyelectrolyte complexation methods. Recently, we have reported the preparation of nanoparticles highly loaded with a granulocyte colony-stimulating factor (GCSF) protein. The applied polymers were either chitosan grafted with PGlu chains [65] or PGlu modified with octyl hydrophobic substituents [66]. Complementary to study the effect of hydrophobic substituent of PGlu [66], our interest is to evaluate the effect of saccharide substituents on the interactions with GCSF protein and, consequently, nanoparticle formation. 

Herein, we report the modification of PGlu carboxyl side groups with glucosamine hydrochloride (GlcN·HCl) using DMTMM as a coupling reagent which does not require the use of protection/deprotection chemistry for the saccharide’s hydroxyl groups. The resulting copolymers P(Glu-GlcN) have been characterized by 1D and 2D NMR techniques to elucidate their detailed structure and composition and by SEC-MALS to determine the copolymers' molar mass averages and molar mass distribution.

## 2. Results and Discussion

**Scheme 1:**
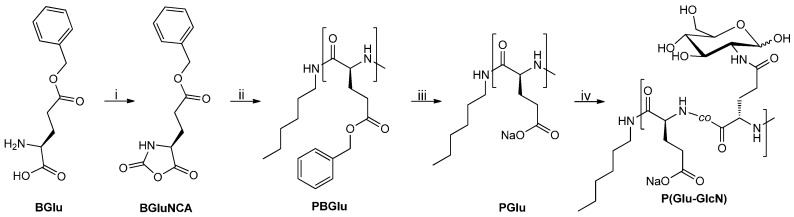
Synthetic pathway for the preparation of P(Glu-GlcN) copolymers.

**Figure 1:**
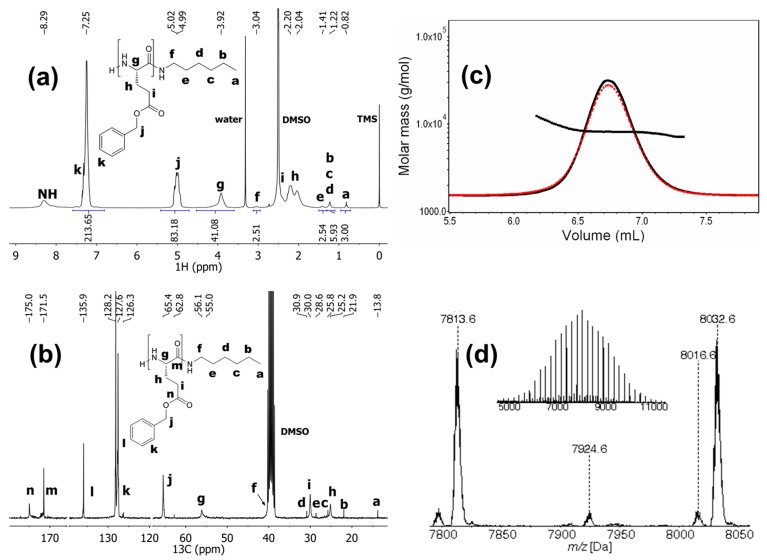
(**a**) ^1^H-NMR spectrum of PBGlu recorded in DMSO-*d*_6_. (**b**) ^13^C-NMR spectrum of PBGlu recorded in DMSO-*d*_6_. (**c**) Enlarged SEC-MALS chromatogram of PBGlu (solid line: RI response, dashed line: LS response at angle 90°) together with the molar mass *vs.* elution volume. (**d**) Enlarged MALDI-TOF mass spectrum of PBGlu at the peak apex, recorded in reflector positive ion mode. Molecular weights are annotated. Inset shows wider mass range of PBGlu mass spectrum.

BGlu NCA monomer was synthesized from BGlu starting material using triphosgene in dry THF (Scheme 1). The BGlu NCA was purified by recrystallization. ROP of BGlu NCA was initiated by hexylamine in dry DMF and the reaction mixture was kept in an ice-bath (Scheme 1) [67]. The conversion of BGlu NCA was monitored by disappearance of the signal for the NCA –NH–group at 9.1 ppm in the ^1^H-NMR spectra of reaction aliquots. When the NCA was consumed, the reaction mixture was precipitated in ice-cold deionized water, the product collected by centrifugation and freeze-dried. The PBGlu was obtained in 94% yield and was characterized by ^1^H- and ^13^C-NMR spectroscopy, SEC-MALS and MALDI-TOF MS to confirm the proposed structure and to determine the molar mass characteristics (Figure 1).

*Reagents and conditions*: (i) Triphosgene, THF, 55 °C; (ii) [NCA]_0_:[hexylamine]_0_ = 50:1, DMF, 0 °C; (iii) HBr/AcOH, TFA, r.t., dialysis; (iv) DMTMM, GlcN·HCl (in three portions), water, pH 8, r.t., dialysis.

**Figure 2:**
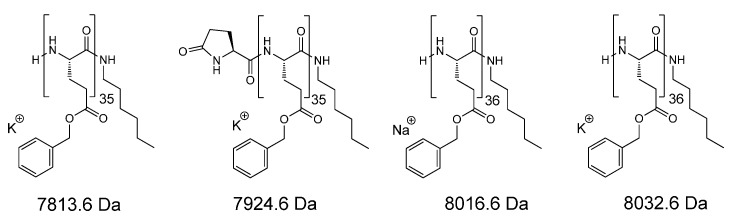
Structures of the species as assigned from the PBGlu MALDI-TOF mass spectrum (Figure 1d).

The number-average degree of polymerization (*DP*_n_) of PBGlu was 41, as determined by ^1^H-NMR spectroscopy by comparing the integrals of the signals for the α-proton of BGlu moiety (signal *g*, 3.92 ppm) and the methyl group of hexylamine initiator moiety (signal *a*, 0.82 ppm), (Figure 1a). SEC-MALS chromatogram shows narrow molar mass distribution of the PBGlu homopolymer with *Đ*_M_ of 1.06 and the *M*_n_ of 8.4 kDa that corresponds to the *DP*_n_ of 38 (Figure 1c). The peak apex in MALDI-TOF mass spectrum of the PBGlu homopolymer belongs to the 36-mer and shows, beside the desired species with unmodified N-terminus and hexylamine C-terminus (signals A and C; potassium and sodium adducts), the presence of the species with the pyrrolidone and hexylamine end groups (signal B; potassium adduct), which are formed by intramolecular cyclization of the amino end group with the adjacent benzyl ester (Figure 1d). The structures of the corresponding species identified from the PBGlu MALDI-TOF mass spectrum are presented in Figure 2. 

**Figure 3:**
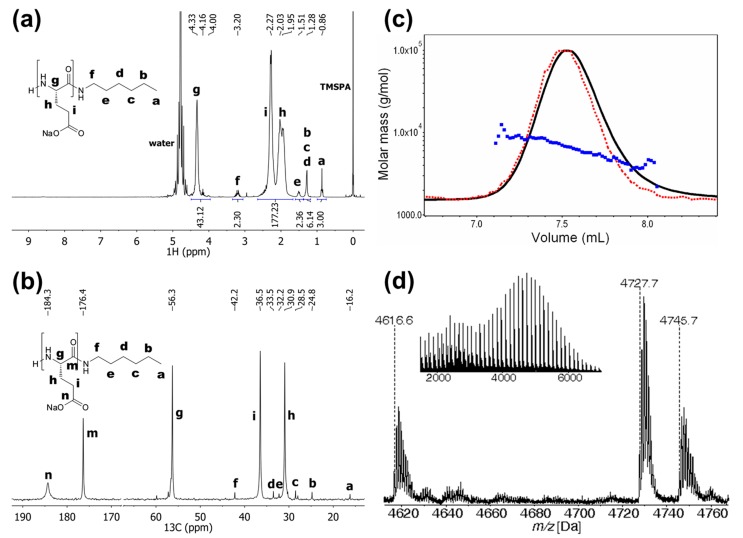
(**a**) ^1^H-NMR spectrum of PGlu recorded in D_2_O. (**b**) ^13^C-NMR spectrum of PGlu recorded in D_2_O. (**c**) Enlarged SEC-MALS chromatogram of PGlu (solid line: RI response, dashed line: LS response at angle 90°) together with the molar mass *vs.* elution volume. (**d**) Enlarged MALDI TOF mass spectrum of PGlu at the peak apex, recorded in reflector negative ion mode. Monoisotopic molar masses are annotated. Inset shows wider mass range of PGlu mass spectrum.

PBGlu was deprotected by applying HBr/acetic acid in TFA (Scheme 1). The obtained product was dissolved in saturated NaHCO_3_, purified by dialysis, and finally freeze-dried to obtain PGlu as sodium salt. PGlu was characterized by ^1^H- and ^13^C-NMR experiments, SEC-MALS and MALDI-TOF MS (Figure 3). 

**Figure 4:**
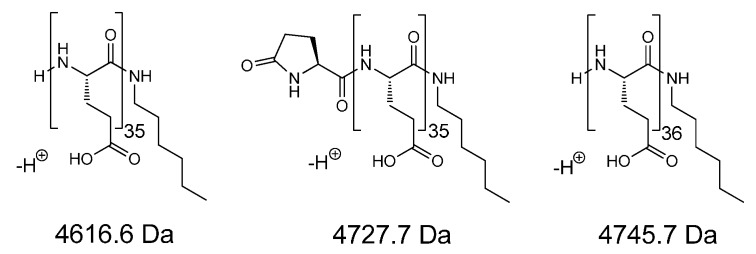
Structures of the species as assigned from the PGlu MALDI-TOF mass spectrum (Figure 3d).

According to the ^1^H-NMR spectrum, the PGlu is quantitatively deprotected. The *DP*_n_ determined by ^1^H-NMR was calculated by comparing the integrals of the signals for the α-proton of the Glu moiety (signal *g*, 4.33 ppm) and the methyl group of the hexylamine initiator moiety (signal *a*, 0.86 ppm) (Figure 3a). Thus obtained PGlu *DP*_n_ is 43 and is in good agreement with the *DP*_n_ value of 42 determined by SEC-MALS (Figure 3c). Similar as MALDI-TOF mass spectrum of PBGlu, the mass spectrum of PGlu (Figure 3d) reveals the species corresponding to the PGlu chains with the unmodified amine N-terminus and the hexylamine C-terminus (signal D) as well as the PGlu species with the pyrrolidone and the hexylamine end groups (signal E). The peak apex belongs to the 36-mer as that in the mass spectrum of the benzyl-protected form (PBGlu). The structures of the corresponding species identified from the PGlu MALDI-TOF mass spectrum are presented in Figure 4.

**Scheme 2:**
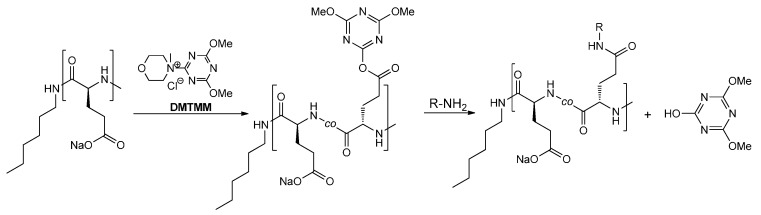
Reaction scheme of DMTMM promoted amide coupling of GlcN and PGlu [64,68].

A post-polymerization modification to prepare the P(Glu-GlcN)s with different degree of GlcN functionalization and most probably randomly distributed GlcN moieties along the PGlu backbone was conducted in aqueous solution by portion-wise addition of DMTMM and GlcN.HCl reagents to the PGlu aqueous solution (Scheme 1 and Scheme 2). The pH of the reaction mixture was adjusted after every addition of the reagents to 8 by the addition of saturated NaHCO_3_ solution. The reaction mixture was stirred for 24 h. 

The degree of substitution with GlcN (*DG*) was altered by the amounts of GlcN·HCl and DMTMM coupling reagent added. The GlcN·HCl was added in two-times molar excess relative to the DMTMM reagent (Table 1). The obtained P(Glu-GlcN) copolymers were purified by dialysis. The P(Glu-GlcN) copolymers were characterized by NMR experiments in D_2_O and SEC-MALS using alkaline aqueous mobile phase (pH 10). The 45% P(Glu-GlcN) copolymer was additionally characterized in-depth by ^13^C, COSY and gHSQCad NMR experiments in D_2_O.

**Table 1:** ^1^H-NMR and SEC-MALS results for the P(Glu-GlcN) copolymers.

Sample Denotation	Reaction Conditions	^1^H-NMR		(*M*_n_)_calc._ (kDa)	SEC MALS			Yield (%)
	Glu:GlcN:DMTMM	*DG* (%)	*DS*_(triazinyl)_ (%)		*M*_n_ (kDa)	*M*_w_ (kDa)	*Đ*_M_	
**PGlu**	-	-	-	-	6.4	6.9	1.1	95
**14% P(Glu-GlcN)**	1:0.5:0.25	14	1	7.5	7.4	9.2	1.2	87
**23% P(Glu-GlcN)**	1:1:0.5	23	2	8.1	8.0	9.5	1.2	74
**45% P(Glu-GlcN)**	1:1.5:0.75	45	3	9.3	8.9	9.9	1.1	76

(*M*_n_)_calc._ was calculated from the *DS*_(GlcN)_ as determined from the ^1^H-NMR spectra of the P(Glu-GlcN) copolymers and the PGlu DP_n_ of 43. *DG* in mol % is the degree of carboxyl group modification with GlcN. *DS* in mol % is the residual degree of substitution with triazinyl activated ester.

**Figure 5:**
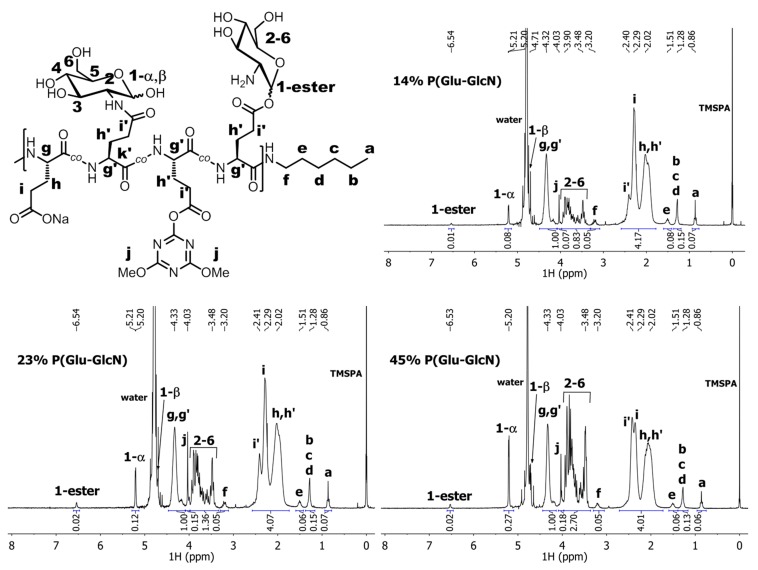
^1^H-NMR spectra of P(Glu-GlcN) copolymers recorded in D_2_O.

*DG* was determined by ^1^H-NMR (Figure 5) by normalizing the integral value of the signal for the PGlu α-CH proton to one (signal *g*, assigned to the α-CH proton of the unmodified Glu repeat units, and signal *g'*, assigned to the α-CH proton of the modified Glu repeat units; both integrated for one proton at 4.32 ppm). 

**Figure 6:**
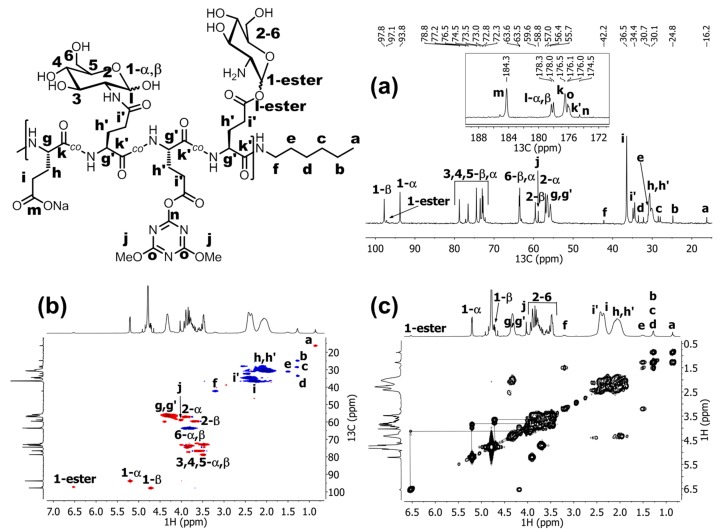
(**a**) ^13^C-NMR spectrum of 45% P(Glu-GlcN). (**b**) gHSQCad NMR spectrum of 45% P(Glu-GlcN). (**c**) COSY NMR spectrum of 45% P(Glu-GlcN). All NMR spectra were recorded in D_2_O.

The proton signals *g* and *g'* overlap, but the ^13^C- and gHSQCad NMR data (Figure 6) distinctly show the presence of two different signals for the corresponding carbon atoms. Then, a set of the signals belonging to GlcN substituents (signals *2–6*, six protons between 3.48–3.94 ppm) was integrated and the obtained integral value was divided by six to determine the molar ratio between the GlcN substituent and the Glu backbone units. All three P(Glu-GlcN) samples show the *DG* of approximately one half of the targeted value (Table 1).

In the ^1^H-NMR spectra of the P(Glu-GlcN) copolymers (Figure 5) a singlet *j* was observed at 4.03 ppm that corresponds to the OMe groups of the 4,6-dimethoxy-1,3,5-triazin-2-yl-activated ester moiety (Scheme 2), which were not aminolyzed by GlcN. Residual degree of substitution with triazinyl activated ester (*DS*_(triazinyl)_) was determined by dividing the integral value of the methoxy signal (signal *j*, six protons at 4.03 ppm) by six. The percent of residual triazinyl active ester moiety present in P(Glu-GlcN) copolymers was determined to be 1, 2 and 3 mol % for the 14% P(Glu-GlcN), 23% P(Glu-GlcN) and 45% P(Glu-GlcN) copolymers, respectively (Table 1). The presence of the dimethoxy triazinyl ester moiety in P(Glu-GlcN) copolymers further confirms the ^13^C- and gHSQCad NMR spectra (Figure 6a,b) for the 45% P(Glu-GlcN)). In the ^13^C-NMR spectrum the signals at 58.8, 174.5 and 176.1 ppm are assigned to the methoxy carbon *j*, the triazine ring carbon *n* and the triazine ring carbons *o*, respectively (Figure 6a) [68], whereas the gHSQCad spectrum confirms the correlation between the methoxy protons signal *j* at 4.03 ppm and the corresponding carbon signal at 58.82 ppm (Figure 6b). 

Additionally, a weak signal at relatively high chemical shift (*1-ester* signal, one proton at 6.54 ppm) was observed in the ^1^H-NMR spectra of P(Glu-GlcN), which indicates a small degree of a side-product formation, *i.e.*, the *1-ester* signal due to the anomeric proton of GlcN substituent which is coupled to a Glu residue by an ester bond in position 1. The percent of the ester-coupled GlcN side-product relative to the Glu backbone residues was determined to be 1, 2 and 2 mol % for the 14% P(Glu-GlcN), 23% P(Glu-GlcN) and 45% P(Glu-GlcN) copolymers, respectively. The presence of the ester coupled GlcN substituents is further confirmed by the gHSQCad (Figure 6b) and COSY (Figure 6c) NMR spectra. In COSY spectrum a cross-peak (at 6.54 ppm, 4.13 ppm) is noticed and in gHSQCad the *1-ester* anomeric proton signal (6.54 ppm) is correlated to the carbon signal at 97.2 ppm. This *1-ester* carbon signal at 97.2 ppm lies between *1-*α and *1-*β carbon signals of the amide coupled GlcN substituents. Large downfield shift of the *1-ester* anomeric proton signal is a consequence of coupling reaction with 1-OH of GlcN rather than –OH groups in other position (3, 4 or 6) since anomeric hydroxyl group is more reactive due to its lower pK_a_ value [69,70,71,72].

It should be noted that complete hydrolysis of the active ester moiety residues of P(Glu-GlcN) copolymers can be achieved by treatment of the samples with 10% Na_2_CO_3_ aqueous solution, however, in addition to accompanying hydrolysis of the ester-conjugated GlcN substituents, the resulting P(Glu-GlcN) copolymers suffered from severe polypeptide backbone degradation as indicated by SEC-MALS (results not shown). The signals of the activated triazinyl ester moiety (signal *j*, 4.03 ppm) and the *1-ester* signal at 6.54 ppm) can be observed also in the NMR spectrum of the analogous product reported by Mildner and Menzel [52]. 

**Figure 7:**
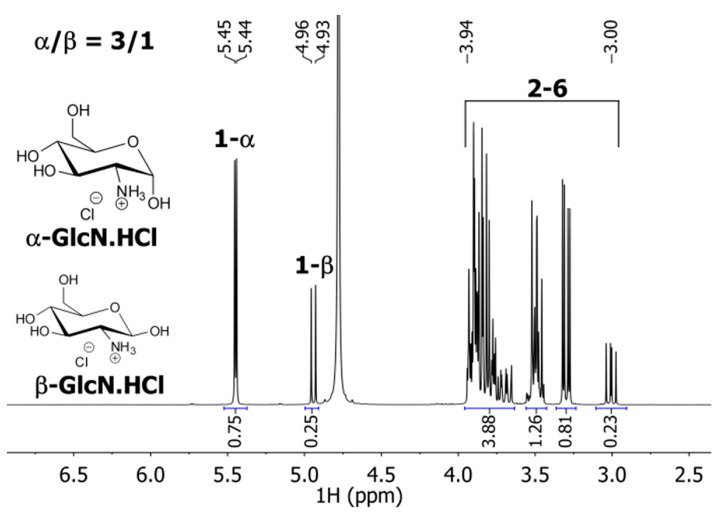
^1^H-NMR spectrum of GlcN.HCl recorded in D_2_O.

The ratio between the α- and β-form of GlcN substituent coupled to Glu repeat units by the amide bond was also estimated. Since the *1-*β signal at ~4.7 ppm in the ^1^H-NMR spectra of P(Glu-GlcN) copolymers (Figure 5) overlaps with the solvent signal, the proportion of the β-form was determined indirectly from the *DG* value (determined from the integral values of the *2–6* signals as described before), which was diminished by the integral values of *1-ester* and *1-α* signals. The *1-α* integral value was then divided by the calculated *1-*β value, and finally, the α/β ratios of 1.6/1, 1.3/1 and 1.7/1 for the 14% P(Glu-GlcN), 23% P(Glu-GlcN) and 45% P(Glu-GlcN), respectively, were determined. For comparison, the ^1^H NMR spectrum (recorded in D_2_O, 25 °C) of the starting GlcN.HCl material (Figure 7) reveals the ratio between the α and β GlcN form of 3/1. The presence of the signal for the GlcN *1-*β anomeric proton is revealed in the gHSQCad (Figure 6b) and COSY (Figure 6c) spectra. 

**Figure 8:**
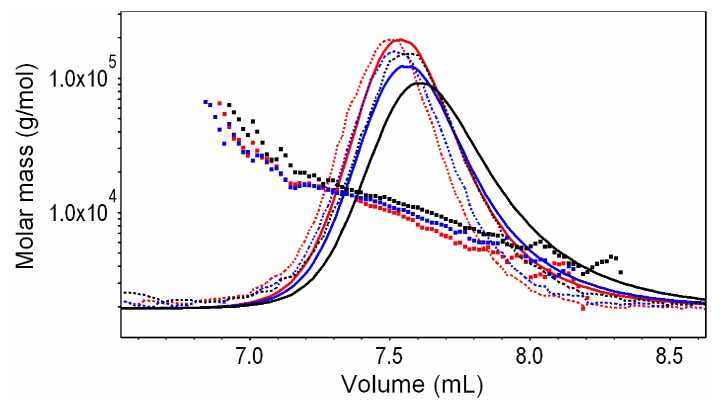
Enlarged SEC-MALS chromatograms (solid lines: RI responses, dashed lines: LS responses at angle 90°) of P(Glu-GlcN) copolymers: 14% P(Glu-GlcN): blue curve, 23% P(Glu-GlcN): red curve and 45% P(Glu-GlcN): black curve, together with molar mass *vs.* elution volume.

The number-average molar masses ((*M*_n_)_calc._) of the synthesized glycopolypeptides were calculated from the P(Glu-GlcN) ^1^H-NMR spectra based on the actual *DG* determined from the proton NMR spectra and the PGlu *DP*_n_ of 43. The (*M*_n_)_calc._ values are in good agreement with the *M*_n_ values determined by SEC-MALS (Table 1, Figure 8), indicating that under the applied experimental conditions the DMTMM coupling is a mild synthetic procedure which negligible impacts the polypeptide backbone degradation. In SEC-MALS chromatograms of P(Glu-GlcN) copolymers the peak slightly shifts toward larger elution volume with increasing molar mass, whereas the relative intensity of the light-scattering signal increases in the same order (Figure 8). These results indicate a change in P(Glu-GlcN) hydrodynamic volume with increasing the *DG*.

## 3. Experimental Section 

### 3.1. Materials

All solvents and reagents were used as received. Dry tetrahydrofuran (THF, anhydrous, >99.9%), dry *N*,*N*-dimethylformamide (DMF, anhydrous, 99.8%) and trifluoroacetic acid (TFA, 99%) were obtained from Sigma-Aldrich (St. Louis, MO, USA). THF (p.a.), *n*-hexane (p.a.), diethyl ether (p.a.) and NaHCO_3_ (>99.0%) were obtained from Merck (Darmstadt, Germany). γ-Benzyl-L-glutamate (BGlu) (>99%), DMTMM (99+%) and hydrogen bromide (pure, 33 wt.% solution in glacial acetic acid) (HBr/AcOH) were obtained from Acros Organics (Geel, Belgium). Triphosgene (>99.0%) and *n*-hexylamine (99%) were obtained from Aldrich. D-(+)-Glucosamine hydrochloride (GlcN.HCl) (>99%, BioReagent) was obtained from Sigma (Steinheim, Germany). NaCl (Ph Eur) was obtained from Fluka (Buchs, Switzerland). Matrix and cationizing agent used for MALDI-TOF MS analysis, super DHB, which is a 9/1 (w/w) mixture of 2,5-dihydroxybenzoic acid and 2-hydroxy-5-methoxybenzoic acid, and sodium trifluoroacetate (NaTFA), respectively, were both obtained from Sigma-Aldrich.

### 3.2. Methods

#### 3.2.1. NMR

^1^H-, ^13^C-, ^1^H-^1^H Correlation Spectroscopy (COSY) and ^1^H-^13^C gradient Heteronuclear Single Quantum Coherence adiabatic version (gHSQCad) spectra were recorded in DMSO-*d*_6_ or in D_2_O on a Unity Inova 300 instrument (Varian, Palo Alto, CA, USA) in the pulse Fourier-transform mode with a relaxation delay of 5 s, and an acquisition time of 3 s. Tetramethylsilane (TMS, δ = 0) and 3-trimethylsilyl-2,2',3,3'-d4-propanoic acid sodium salt (TMSPA, δ = 0) were used as an internal chemical-shift standards in DMSO-*d*_6_ and D_2_O, respectively.

#### 3.2.2. Size-Exclusion Chromatography Coupled to a Multi-Angle Light-Scattering Detector (SEC-MALS)

The SEC-MALS measurements were performed using an isocratic pump with an online vacuum degasser and an autosampler (all Agilent 1260 type, Agilent Technologies, Santa Clara, CA, USA). The separations of samples were carried out on a PolarGel-L column (Agilent Technologies, molar mass range: up to 30 kDa). Two different eluents were used: (i) 0.1 M sodium nitrate (NaNO_3_) solution with sodium azide (NaN_3_) (0.02% w/v) (both from Sigma-Aldrich), prepared with MiliQ water (18.2 MΩ/cm) at pH 10 for PGlu and P(Glu-GlcN) samples; and (ii) *N*,*N*-dimethylacetamide (DMAc) with 0.05 M lithium bromide (LiBr) (both from Sigma-Aldrich) for PBGlu sample. The detection was done through a system of successively on-line connected detectors: a multi-angle light-scattering DAWN-HELEOS detector (MALS with 18 angles), operating at a wavelength of 658 nm, and an interferometric refractive index (RI) detector Optilab rEX (both instruments from Wyatt Technology Corporation, Santa Barbara, CA, USA), operating at the same wavelength as the MALS detector. The nominal eluents flow rate was 1.0 mL/min. The mass of the samples injected onto the column was typically 1 × 10^−4^ g, whereas the solution concentration was 1 × 10^−3^ g/mL. The determination of absolute Mw and the calculation of *M*_n_ values from MALS detector require a sample-specific refractive-index increment (d*n*/d*c*), which was determined from the RI response assuming 100% of sample mass recovery from the column. For the data acquisition and evaluation Astra 5.3.4 software (Wyatt Technology Corporation) was utilized.

#### 3.2.3. Matrix-Assisted Laser Desorption/Ionization Time-of-Flight Mass Spectrometry (MALDI-TOF MS)

The MALDI-TOF MS measurements were performed with an UltrafleXtreme MALDI-TOF-TOF mass spectrometer (Bruker Daltonik, Bremen, Germany) equipped with a 337 nm nitrogen laser, capable of executing reflector mode analysis. The reflector positive ion mode was used for acquiring the mass spectra of PBGlu and the reflector negative ion mode for the mass spectra of the PGlu. Dried-droplet method was used to spot the samples on a MALDI plate. The solutions of PBGlu (2 mg/mL), super DHB (20 mg/mL) and NaTFA (5 mM) in THF were mixed in a volume ratio of 1/10/1. PGlu was dissolved in water (2 mg/mL) and mixed with a solution of super DHB (20 mg/mL) in 30/70 (v/v) mixture of acetonitrile/aqueous 0.1% TFA (volume ratio: 1/10). In all cases 0.5 µL of the mixture was deposited on a MALDI target and allowed to dry on air. The mass spectra were acquired by summing spectra from ~500 selected laser shots. The calibration was made externally with a Peptide calibration standard II and Protein Calibration Standard I (Bruker Daltonik). The data were processed with Bruker FlexAnalysis 3.3.80 software.

### 3.3. Synthesis

#### 3.3.1. γ-Benzyl-L-glutamate NCA (BGlu NCA)

A 250 mL flame-dried round-bottom flask was charged with BGlu (6.2 g, 26 mmol) and triphosgene (3.7 g, 13 mmol) under argon. Then, dry THF (130 mL) was added and the reaction mixture was stirred at 55 °C for 90 min. The clear reaction mixture was then concentrated under vacuum followed by precipitation in hexane. The product was crystallized three times from THF/hexane. Yield: 6.1 g (89%).

#### 3.3.2. Poly(γ-benzyl-L-glutamate) (PBGlu)

BGlu NCA (5.0 g, 19 mmol) was dissolved in dry DMF (62 mL) under argon in an ice-bath. A solution of hexylamine (0.38 mmol) in dry DMF (1 mL) was added and the reaction mixture was stirred in ice-bath for 2 days. Then, the reaction mixture was precipitated into cold deionized water. The product was collected by centrifugation, washed with water several times and freeze-dried to obtain white powdery material. Yield: 3.9 g (94%). SEC-MALS: *M*_n_ = 8.4 kDa, *M*_w_ = 8.9 kDa, *Đ*_M_ = 1.06.

#### 3.3.3. Poly(sodium-L-glutamate) (PGlu)

PBGlu (1.0 g, 4.7 mmol) was dissolved in TFA (25.6 mL) under argon. HBr/AcOH (33 wt.%) (3.24 mL, 18.7 mmol) was slowly added. Reaction mixture was stirred for 90 min at room temperature under argon. The deprotected polymer was precipitated in cold diethyl ether and collected by centrifugation. The product was dissolved in saturated NaHCO_3_, dialyzed (MWCO: 100–500 Da) against deionized water and finally freeze-dried to obtain PGlu as sodium salt. Yield: 0.67 g (95%). Debenzylation: quantitative. SEC-MALS: *M*_n_ = 6.4 kDa, *M*_w_ = 6.9 kDa, *Đ*_M_ = 1.08.

#### 3.3.4. General Procedure for Modification of PGlu with GlcN (P(Glu-GlcN))

PGlu (1 mmol Glu units) was dissolved in deionized water (10 mL). The DMTMM and GlcN.HCl were added in three portions (one portion on three hours). The pH of the reaction mixture was adjusted to 8 by addition of saturated NaHCO_3_ after every addition of GlcN.HCl. The reaction mixture was stirred at room temperature for another 18 h. Then, it was transferred to dialysis bag (MWCO: 100–500 Da) and dialyzed against 0.1 M NaCl and against deionized water. After dialysis, the product was freeze-dried to obtain white fluffy solid. The ratio between PGlu, DMTMM and GlcN.HCl for the synthesis of differently substituted P(Glu-GlcN), the reaction yields and the molar mass characteristics of the products as determined by SEC-MALS are given below:

*14% P(Glu-GlcN)*: PGlu (1 mmol Glu units), DMTMM (0.25 mmol), GlcN.HCl (0.5 mmol). Yield: 161 mg (87%). SEC-MALS: *M*_n_ = 7.4 kDa, *M*_w_ = 9.2 kDa, *Đ*_M_ = 1.2.
*23% P(Glu-GlcN)*: PGlu (1 mmol Glu units), DMTMM (0.50 mmol), GlcN.HCl (1.0 mmol). Yield: 164 mg (74%). SEC-MALS: *M*_n_ = 8.0 kDa, *M*_w_ = 9.5 kDa, *Đ*_M_ = 1.2.
*45% P(Glu-GlcN)*: PGlu (1 mmol Glu units), DMTMM (0.75 mmol), GlcN.HCl (1.5 mmol). Yield: 193 mg (76%). SEC-MALS: *M*_n_ = 8.9 kDa, *M*_w_ = 9.9 kDa, *Đ*_M_ = 1.1.

## 4. Conclusions

DMTMM was evaluated as a coupling reagent in post-polymerization modification of PGlu with GlcN to prepare P(Glu-GlcN) copolymers. Detailed characterization of the obtained P(Glu-GlcN) copolymers by 1D and 2D NMR experiments has shown successful functionalization of PGlu with GlcN via amide bond formation. The GlcN substituents were coupled to PGlu by the ester bond in position 1 to negligible extent. Additionally, up to 3 mol % of residual dimethoxytriazinyl active ester moiety were found to be present in all the obtained P(Glu-GlcN) copolymers. SEC-MALS results indicate negligible degradation of the polypeptide backbone during the glycosylation reaction, proving that DMTMM is a mild and effective amidation reagent.
